# Efficacy and safety of intravenous thiamylal in sedation for colonoscopy in children

**DOI:** 10.1002/deo2.70022

**Published:** 2024-09-29

**Authors:** Sotaro Ozaka, Haruhiko Takahashi, Yuta Shimomori, Yomei Kagoshima, Shohei Terashi, Koshiro Tsutsumi, Ryota Sagami, Yuka Hirashita, Kensuke Fukuda, Ryo Ogawa, Masaaki Kodama, Kazunari Murakami, Kazuhiro Mizukami

**Affiliations:** ^1^ Department of Gastroenterology Faculty of Medicine Oita University Oita Japan

**Keywords:** children, colonoscopy, midazolam, sedation, thiamylal

## Abstract

**Objectives:**

Since a standard sedation protocol for pediatric colonoscopy (CS) has not been established, evidence on optimal sedative agents is needed. This study aimed to evaluate the efficacy and safety of thiamylal in sedation for pediatric CS compared to midazolam.

**Methods:**

Children from 7 to 16 years of age who underwent CS under sedation with intravenous thiamylal or midazolam at our hospital between June 2010 and March 2024 were included in this retrospective observational study. The primary outcome was the efficacy (success rate of CS without mid‐awakening) of the drugs. Meanwhile, the secondary outcomes were the sedation level during CS, procedure time, recovery time, and adverse events related to sedation.

**Results:**

Sixty children were included in the study. The success rate of CS without mid‐awakening was significantly higher in the thiamylal group (90.6%) than in the midazolam group (64.3%; *p* = 0.03). The two groups had no significant differences in median sedation depth, procedure time, or recovery time. Adverse events related to sedation in thiamylal group (22%) and midazolam group (25%) were similar. No severe adverse events were reported.

**Conclusions:**

Intravenous thiamylal provides effective and safe sedation in children requiring CS, with little or no mid‐awakening during the procedure.

## INTRODUCTION

With the increasing number of patients with inflammatory bowel disease and allergic gastrointestinal disorders, there has been an increase in cases requiring colonoscopy (CS) during childhood.^1^, ^2^ Appropriate sedation should be provided when performing gastrointestinal endoscopy to ensure the examination is performed safely and painlessly. However, children are often unable to follow an examiner's instructions or undergo CS with self‐control, and inadequate sedation causes physical and emotional distress to both patients and endoscopists. While adult patients undergo CS with conscious sedation, pediatric patients require deep sedation because of their relatively high level of anxiety, lack of cooperation, and pain sensitivity.^3^, ^4^ Some studies have shown that conscious sedation in a considerable percentage of children results in an unsafe, poor‐quality, prolonged, or aborted CS.^5^ It is also important to pay attention to the side effects of sedation, such as respiratory and cardiovascular complications. However, a standard sedation protocol for pediatric colonoscopies has not yet been established.

At our facility, endoscopists and pediatricians collaborate to provide CS to children. We used midazolam and thiamylal for endoscopic sedation because of their rapid effects and safety profile. Since 2010, we have used intravenous thiamylal effectively and safely for endoscopic sedation in pediatric patients. Thiamylal is a barbiturate sedative used in pediatric patients undergoing magnetic resonance imaging and other orthostatic procedures.^6^, ^7^, ^8^ Few facilities use it for the sedation of pediatric CS, and there are no reports on its efficacy and safety. We hypothesized that thiamylal would have a better sedative effect than midazolam because of its shorter duration of sedation and reduced mid‐awakening. Therefore, this pilot study aimed to compare the efficacy and safety of intravenous thiamylal and midazolam for sedation during pediatric CS.

## METHODS

### Participants

This retrospective observational study was based on a single‐facility database. Pediatric patients who underwent CS at the Oita University Hospital between June 2010 and March 2024 were included in the study. Children aged 7–16 years who received CS sedation in the endoscopy room were enrolled in the study. The exclusion criteria were proctosigmoidoscopy, CS without sedation, CS with general anesthesia, and inadequate data. The group sedated with thiamylal alone or in combination with ketamine was defined as the thiamylal group, whereas the group sedated with a combination of midazolam and pethidine was defined as the midazolam group.

### Study design

Using electronic medical record data, we retrospectively analyzed patient characteristics, including age, sex, body weight, and American Society of Anesthesiologists (ASA) physical status classification (ASA class I, a normal healthy patient; ASA class II, a patient with mild systemic disease; ASA class III, a patient with severe systemic disease). In all cases, written informed consent was obtained from at least one parent before endoscopy. The patients underwent CS alone or combined endoscopy (CS and esophagogastroduodenoscopy [EGD]). Patients who underwent combined endoscopy underwent EGD followed by CS. Bowel‐cleaning preparations for CS, such as magnesium citrate, polyethylene glycol, and polyethylene glycol plus ascorbic acid, licensed in Japan, were selected by pediatricians based on age, body weight, and clinical state. Sedation was usually performed by at least two pediatricians, and CS was performed by experienced endoscopists. The choice of sedative medication was made by the pediatrician responsible for sedation. Midazolam is the drug of choice for children with a history of bronchial asthma. The analgesics included ketamine in the thiamylal group and pethidine in the midazolam group. Anesthesia was induced with thiamylal (3–4 mg/kg), ketamine (1 mg/kg), midazolam (0.05 mg/kg), and pethidine (35 mg). In the thiamylal group, atropine (10 mg) was administered at the start of anesthesia to prevent a ketamine‐induced increase in airway secretion. Drug doses were determined based on Japanese guidelines.^9^ Sedation level was assessed using the Pediatric Sedation State Score (PSSS; Table S1).^10^

After administering anesthetics, achievement of PSSS <4 was confirmed, and the examination was started. Procedure time was defined as the time from insertion to endoscope removal. The pediatrician regularly monitored and recorded the heart rate, SpO2, and blood pressure during the procedure. Hypotension, bradycardia, and hypoxemia were also observed. Additional adverse events, including vomiting, agitation, and hyperventilation, were recorded. Hypoxemia was considered when SpO_2_ is < 92%. No routine oxygen therapy was administered. The patients were evaluated for PSSS every 5 min from drug administration until the end of endoscopy. Because the patient was considered apt to tolerate CS without body movement requiring restraint, we defined adequate sedation if the PSSS score was < 4. If there was any movement or decrease in sedation depth during the endoscopy, an additional dose of thiamylal (1–2 mg/kg) or midazolam (0.025–0.05 mg/kg) was administered. Mid‐awakening was defined as the persistence of scores of 4 or higher on the PSSS, even after an additional dose. After the endoscopic procedure, the patient was transferred to an observation room and monitored by a nurse until fully awake. Adverse events during the recovery period were recorded. Full awakening from sedation was defined according to our institution's observation room exit criteria as follows: (1) patient can respond clearly to the call; (2) patient can move arms and legs freely and walk without wobbling; (3) patient can breathe deeply and cough freely; (4) systolic blood pressure >100 mmHg or recovered to the value immediately before sedation; (5) SpO2: 92% or higher without oxygen administration; and (6) no restless or erratic behavior. Recovery time was defined as the time from the end of the CS to full awakening.

### Outcomes

The primary outcome was the success rate of CS without mid‐awakening. Successful CS was defined as observing the total colon without interrupting the procedure. Secondary outcomes were the sedation level during endoscopy, procedure time, recovery time, and sedation‐related adverse events.

### Statistical analysis

Data were analyzed using EZR software version 1.54. We examined the difference between the thiamylal and midazolam groups using the Mann–Whitney *U* test to compare the median values of non‐normally distributed variables and Pearson's chi‐square test or Fisher's exact test for nominal variables. The difference between the study groups was considered statistically significant when the *p*‐value was <0.05.

### Ethical consideration

This study was conducted in accordance with the Declaration of Helsinki (revised in 2013). This study was approved by the Institutional Review Board at Oita University (IRB No. 2793). Patients and parents were fully informed about the sedation. Written informed consent was obtained from at least one parent.

## RESULTS

### Patient characteristics

**FIGURE 1:**
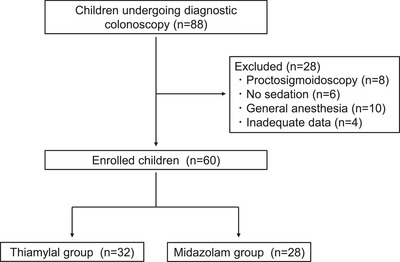
A flow chart of the present study.

Colonoscopies were performed in 88 patients during the study period. After excluding 28 patients (eight proctosigmoidoscopy, six without sedation, 10 with general anesthesia, and four with inadequate data), 60 patients were enrolled. A final analysis was performed as available case analysis on 32 patients in the thiamylal group and 28 in the midazolam group (Figure 1). Baseline patient characteristics are shown in Table 1. The median age of participants was 14 years. The median age was significantly lower in the thiamylal group than in the midazolam group (*p* < 0.01).

**TABLE 1:** Patient characteristics.

	All cases (*n* = 60)	Thiamylal group (*n* = 32)	Midazolam group (*n* = 28)	*p*‐value
Age, years, median (interquartile range)	14 (12–16)	12 (11–13)	15 (14–16)	<0.01
Male, *n* (%)	36 (60)	17 (53)	19 (68)	0.37
Weight, kg, median (interquartile range)	43.0 (35.1–51.8)	36.2 (30.3–44.0)	48.0 (45.5–56.8)	<0.01
ASA‐PS, *n* (%)				
I	21 (35)	8 (25)	13 (46)	0.14
II	39 (65)	24 (75)	15 (54)	
Indication of endoscopy^†^, *n* (%)				
Abdominal pain	19 (32)	11 (34)	8 (29)	0.83
Bloody stool	17 (28)	8 (25)	9 (32)	0.74
Diarrhea	20 (33)	10 (31)	10 (36)	0.92
Fever	7 (12)	4 (13)	3 (11)	1.00
Anal lesion	6 (10)	4 (13)	2 (7)	0.68
Screening	8 (13)	4 (13)	4 (14)	1.00
Endoscopic procedures, *n* (%)				
Colonoscopy alone	35 (58)	19 (59)	16 (57)	1.00
Colonoscopy + esophagogastroduodenoscopy	25 (42)	13 (41)	12 (43)	
Sedatives, *n* (%)				
Thiamylal alone	4 (7)	4 (13)		
Thiamylal + ketamine	28 (47)	28 (87)		
Midazolam + pethidine	28 (47)		28 (100)	
ASA‐PS American Society of Anaesthesiologists' physical status classification				

^†^ There is a duplication.

Moreover, the median body weight was significantly lower in the thiamylal group than in the midazolam group (*p* < 0.01). The indications for CS included abdominal pain, bloody stools, diarrhea, fever, anal lesions, and screening. The most common indication for CS was diarrhea (*n* = 20, 33%). There were no significant differences in the indications between the two groups. Combined endoscopy was performed in 42% of patients.

### Efficacy of sedation

The success rates of CS were 100% in the intravenous thiamylal group and 96.4% in the intravenous midazolam group. One patient in the midazolam group required hyperventilation during the CS, and the examination was interrupted. Because mid‐awakening interferes with safe CS,^5^ the success rate of CS without mid‐awakening was analyzed. The success rate of CS without mid‐awakening was significantly higher in the thiamylal group (90.6%) than in the midazolam group (64.3%; *p* = 0.03). The two groups had no statistically significant differences regarding median procedure time, median recovery time, and median PSSS during the procedure (Table 2). The median dose of thiamylal per body weight was 6.46 (4.31–8.71) mg/kg, and midazolam per body weight was 0.11 (0.09–0.15) mg/kg, respectively (Table 2). As body weight is an important factor affecting sedative effects, we performed a subgroup analysis excluding lightweight children. In a subgroup analysis of children aged 13–16 years, the rate of mid‐awakening was significantly lower in the thiamylal group. However, the two groups had no significant difference in body weight (Table 3). The two groups had no significant differences in the median procedure or recovery times.

**TABLE 2:** Results of sedation.

	All cases (*n* = 60)	Thiamylal group (*n* = 32)	Midazolam group (*n* = 28)	*p*‐value
Successful CS, *n* (%)	59 (98.3)	32 (100)	27 (96.4)	0.47
Successful CS without mid‐wakening, *n* (%)	47 (78.3)	29 (90.6)	18 (64.3)	0.03
PSSS, median (interquartile range)	2 (2–3)	2 (2–3)	2.5 (2–3)	0.13
Procedure time (min), median (interquartile range)	31 (21–46)	31 (21–49)	30 (25–44)	0.92
Recovery time (min), median (interquartile range)	38 (20–78)	40 (20–80)	37 (21–60)	0.70
Sedative dose, median (interquartile range)				
Thiamylal (mg/kg)	6.46 (4.31–8.71)	6.46 (4.31–8.71)		
Midazolam (mg/kg)	0.11 (0.09–0.15)		0.11 (0.09–0.15)	
Ketamine (mg/kg)	1.28 (0.99–1.63)	1.28 (0.99–1.63)		
Pethidine (mg/kg)	0.72 (0.62–0.77)		0.72 (0.62–0.77)	
CS colonoscopy, PSSS pediatrics sedation state score				

**TABLE 3:** Subgroup analysis of children aged 13–16 years.

	Thiamylal group (*n* = 18)	Midazolam group (*n* = 28)	*p*‐value
Age, years, median (interquartile range)	13 (13‐16)	15 (14‐16)	0.018
Male, *n* (%)	8 (44)	19 (68)	0.205
Weight, kg, median (interquartile range)	40.9 (35.1‐51.7)	48.0 (45.5‐56.8)	0.105
Mid‐awakening, *n* (%)	1 (6)	10 (36)	0.032
Oxygen desaturation, *n* (%)	4 (14)	3 (17)	1.00

### Adverse events of sedation

The adverse events during the procedure are shown in Table 4. Oxygen desaturation was most frequent, occurring in six patients in the thiamylal group (19%) and four in the midazolam group (14%). All cases were resolved with airway repositioning or temporary oxygen administration. There were no significant differences between the two groups. The two cases of hyperventilation observed in the midazolam group were due to fear. No serious adverse events, such as death, respiratory arrest, laryngospasm, or shock, led to discontinuation of the test. During recovery, vomiting (three children) was observed among the children only in the thiamylal group. Headaches were observed in one patient in each group. All adverse events during the recovery were self‐limited and did not require any medical intervention. No adverse events were observed in all patients at the follow‐up period up to 6 months after sedation.

**TABLE 4:** Adverse events during and after sedation.

	All cases (*n* = 60)	Thiamylal group (*n* = 32)	Midazolam group (*n* = 28)	*p*‐value
Adverse events during sedation, *n* (%)	14 (24)	7 (22)	7 (25)	1.00
Hypoxemia	10 (17)	6 (19)	4 (14)	0.91
Vomiting	1 (2)	1 (3)	0 (0)	
Agitation	1 (2)	0 (0)	1 (4)	
Hyperventilation	2 (3)	0 (0)	2 (8)	
Postsedation adverse events, *n* (%)	5 (8)	4 (13)	1 (4)	0.36
Vomiting	3 (5)	3 (9)	0 (0)	
Headache	2 (3)	1 (3)	1 (4)	

## DISCUSSION

This pilot study demonstrated the efficacy and safety of intravenous thiamylal sedation in pediatric CS compared to intravenous midazolam. Thiamylal demonstrated a 100% CS completion rate and a significantly lower mid‐awakening rate compared to midazolam.

Pediatric gastrointestinal endoscopy has evolved, with increasing diagnostic and therapeutic applications.^11^ However, the painfulness and discomfort of CS make the process difficult for both patients and medical staff.^12^, ^13^ Clinically, most patients report worry before undergoing endoscopy under sedation.^14^ Worries reduce the willingness and compliance with the procedure, leading to delays in diagnosis and treatment.  Therefore, adequate sedation is crucial in pediatric CS because insufficient sedation can lead to severe psychological trauma and serious complications.^15^ Although the guidelines for CS in children have been published by the American Society for Gastrointestinal Endoscopy, North American Society for Pediatric Gastroenterology, Hepatology, and Nutrition, and European Society for Pediatric Gastroenterology Hepatology and Nutrition (ESPGHAN), a consensus protocol for optimum sedation for CS has not been established.^16^, ^17^ Many drugs are available for sedation during pediatric CS. Midazolam is more commonly used in Japan because of its effectiveness and fewer adverse effects.^18^ In addition, because CS is a painful procedure, and sedatives alone provide inadequate sedation, a combination of sedatives and analgesics is recommended.^19^

At our facility, thiamylal and midazolam are used for sedation during CS in children under close monitoring by a pediatrician. Thiamylal is an ultra‐short‐acting barbiturate, similar to thiopental, which exerts a sedative effect by acting as an agonist of GABA_A_ receptors.^20^ Thiamylal has been widely used clinically as an anesthetic agent since around 1950.^21^ Although there have been several reports demonstrating the efficacy and safety of thiamylal in pediatric MRI or other procedural sedation,^6^, ^7^, ^8^ there are few reports on the use of thiamylal for CS in children.  The success rate of CS by sedation with intravenous thiamylal in this study was 100% (Table 2), suggesting significant clinical efficacy. According to a previous report, the midazolam‐ketamine and propofol‐fentanyl combinations have demonstrated a 100% CS success rate in children.^22^ In this study, the combination of thiamylal and ketamine showed a high CS completion rate comparable to that reported in this study. It should be emphasized that patients sedated with intravenous thiamylal had a lower rate of mid‐awakening during CS than those sedated with midazolam. As for midazolam, the combination of midazolam‐fentanyl, midazolam‐ketamine, and midazolam‐meperidine has been reported to have high endoscopy completion rates of 91.6% to 100% and few serious side effects.^23^, ^24^ However, paradoxical reactions are likely to occur with the use of midazolam in young children,^25^ and we often encounter cases of failed induction of sedation or awakening during examination. It is reported that the sedation success rate (PSSS: <3) for midazolam in pediatric MRI was 9.5%, which was significantly lower than the 88.1% rate for dexmedetomidine.^26^

The ESPGHAN suggests performing CS in children under general anesthesia or, if general anesthesia is not available, under deep sedation in a carefully monitored environment.^4^ If a pediatric patient awakens during an endoscopic procedure, they will not be able to control themselves and will have difficulty continuing the examination. This may also lead to psychological trauma in fearful patients. Two patients sedated with midazolam in this study became fearful and hyperventilated, and one had his CS interrupted. Thiamylal may be advantageous over midazolam in maintaining deep sedation with less mid‐awakening. In addition, subgroup analysis showed a significantly higher success rate of CS without mid‐awakening in the thiamylal group among low‐weight children and children with ASA2 comorbidity (Table S2), suggesting that thiamylal may be useful for even low‐weight children who may be anxious about the CS.

In the present study, adverse events during examination occurred in 24% of the cases (Table 3). The most common adverse event was oxygen desaturation, occurring in 10 of 60 patients (17%). Although oxygen desaturation was observed in 6 of 32 patients (19%) in the thiamylal group, the incidence was similar to that in the midazolam group (14%). Irie et al. reported that respiratory complications occurred in 10.2% of pediatric patients who underwent MRI under thiamylal sedation.^6^ Although the incidence of respiratory complications related to thiamylal was more frequent at 19% in this study, this may be due to the longer examination time and higher drug use for endoscopy than for MRI. Importantly, there were no severe adverse respiratory events requiring airway intervention or mask bag ventilation in the present study. In addition, vomiting was observed in 9% (3/32) of patients during recovery. Kinoshita et al. reported vomiting in 22.6% of patients after ablation with thiamylal‐based sedation.^27^

On the other hand, a previous study on midazolam reported that vomiting occurred in 5% of pediatric patients undergoing gastrointestinal endoscopy,^28^ suggesting that vomiting is a relatively common but not unique side effect of thiamylal. Thus, we believe that our sedation protocol using thiamylal is acceptable regarding both the incidence and severity of adverse events. However, according to previous reports, barbiturates are associated with significantly more adverse events than etomidate or propofol.^29^, ^30^ Therefore, sedation using thiamylal should be provided under the strict supervision of a pediatrician skilled in its use.

In this study, thiamylal was used more often in younger children with lighter weight (Table 1). As body weight is an important factor affecting sedative effects, a subgroup analysis was performed, excluding children with lighter body weight. Even in children with higher body weights, the rate of mid‐awakening was significantly lower in the thiamylal group (Table 3). In most cases of CS in adults, the patient can follow the examiner's instructions and can be examined under conscious sedation. Although older children may be able to undergo endoscopy with self‐restraint, some are unable to. Thus, thiamylal may be a good indication for older, larger children and even adults who are uncooperative with endoscopy, who had unsuccessful sedation in the past, or who were anxious and desired deep sedation.

The limitations of this study were as follows. First, it was a retrospective study. There was a tendency for thiamylal to be selected by younger children with low body weights, which could be considered a selection bias. The latest guideline for pediatric gastrointestinal endoscopy in Japan states that it is difficult to recommend a specific drug for sedation.^9^ Therefore, even at our hospital, the criteria were not clearly defined and the choice of sedative was left to the judgment of the anesthesiologist. In our facility, thiamylal tended to be the customary choice for younger children, while midazolam, which is more commonly used in adults, was more likely to be selected for older children. Second, the small sample size precluded the evaluation of the most serious adverse events, such as cardiopulmonary arrest, laryngospasm, incidental aspiration, permanent neurological injury, or death.^31^ Third, not only cases with CS alone but also those with combined EGD (CS + EGD) were included, and sedative doses were probably higher in CS + EGD cases, which are more invasive. Finally, the types of concomitant analgesics used in each group differed, which may be a confounding factor. However, the present findings have important implications for selecting sedatives for pediatric CS. Further prospective clinical studies with sufficient numbers of participants are required to address these limitations.

In conclusion, the results of this study demonstrate the adequate efficacy and safety of intravenous thiamylal for sedation during CS in children. Thiamylal had a higher rate of successful CS without mid‐awakening than midazolam. As there are no previous reports on using thiamylal for sedation in endoscopy, this study provides insights into a new option for sedation in pediatric endoscopy.

## CONFLICT OF INTEREST STATEMENT

None.

## PATIENT CONSENT STATEMENT

Written informed consent was obtained from at least one parent before endoscopy.

## CLINICAL TRIAL REGISTRATION

N/A

## ETHICS STATEMENT

This study was approved by the Institutional Review Board of Oita University Faculty of Medicine, and it was conducted in accordance with the Declaration of Helsinki.

## Supporting information




**TABLE S1** Pediatrics sedation state score (PSSS) is shown.

**TABLE S2** Results of subgroup analysis of sedation effects are shown.

## Data Availability

All pertinent data are included within the paper and its Supporting Information file. The data underlying this article will be shared on reasonable request to the corresponding author.
